# Minimally Invasive Colorectal Cancer Surgery in Europe: Implementation and Outcomes: Erratum

**DOI:** 10.1097/01.md.0000494754.98175.d6

**Published:** 2016-08-26

In the article “Minimally Invasive Colorectal Cancer Surgery in Europe: Implementation and Outcomes”,^1^ which appeared in Volume 95, Issue 22 of *Medicine*, Dr. Lemmens’ affiliation was originally given incorrectly. The article has since been corrected online.
